# Assessing the Efficacy of Lokomat Training in Pediatric Physiotherapy for Cerebral Palsy: A Progress Evaluation

**DOI:** 10.3390/jcm13216417

**Published:** 2024-10-26

**Authors:** Michalina Błażkiewicz, Anna Hadamus

**Affiliations:** 1 Faculty of Rehabilitation, The Józef Piłsudski University of Physical Education in Warsaw, 00-968 Warsaw, Poland; michalina.blazkiewicz@awf.edu.pl; 2 Department of Physiotherapy Fundamentals, Faculty of Dental Medicine, Medical University of Warsaw, 02-091 Warsaw, Poland

**Keywords:** Lokomat, gait therapy, locomotion, stroke, neurorehabilitation, robotic guidance

## Abstract

**Background**: Gait disturbances in children with cerebral palsy can increase the hindrance caused by loss of independence and social engagement. The Lokomat, developed by Hocoma, shows promise as a supplementary tool for gait rehabilitation. This study investigates the impact of Lokomat training on gait parameters and trends observed during training. **Methods**: A total of 26 children (13 male individuals) with a diagnosis of cerebral palsy (CP), aged 4 to 23 years, were enrolled in the study. Patients participated in a standard comprehensive rehabilitation program with additional Lokomat training sessions. Gait function was assessed using the Timed Up and Go Test (TUG) and the 10 m walking test (10mWT) at the beginning and end of the rehabilitation period. Changes in Lokomat parameters (step number, session duration, speed, body weight support, and guidance force) were also analysed. **Results**: The median duration of the 10mWT and TUG significantly decreased across the groups after the treatment program. The highest increases were observed for the number of steps taken. Across the entire cohort, the linear trend curves for distance and number of steps exhibited near-perpendicular alignment with the horizontal axis, suggesting significant improvement in these parameters. A consistent trend was noted for speed, with the trend line aligned parallel with the horizontal axis. Decreasing trends were observed for body weight support and guidance force. **Conclusions**: Therapy with the Lokomat functioning as the active gait orthosis can be used as a form of support to the standard rehabilitation protocol for patients with CP.

## 1. Introduction

Cerebral palsy (CP) encompasses a spectrum of motor impairment and non-progressive postural syndromes emerging in early childhood [1]. Due to abnormal development or damage to the brain areas responsible for muscle control and coordination [2,3], CP is often associated with movement, posture, and balance issues and co-occurs with sensory, cognitive, communication, and behavioural disorders. Hu et al. [2] demonstrated that CP poses a significant public health challenge and imposes a substantial socioeconomic burden worldwide. Hence, the CP issue affects various disciplines, including paediatrics, neurology, paediatric orthopaedics, rehabilitation medicine, orthopaedics, and genetics [4].

Therapy for patients with CP is a lifelong journey. Koman et al. [1] and Mu et al. [5] highlighted that the primary objective of therapy should be its early initiation to enhance functionality and skills while promoting health in multiple domains, such as locomotor function, cognitive development, social integration, and independence. Management strategies cover a spectrum of approaches, including physiotherapy, occupational therapy, and speech therapy. Such strategies also involve applying orthotics, device-assisted modalities, pharmacological interventions, and orthopaedic or neurosurgical procedures [1,6]. Hu et al. [2] showed that an accurate diagnosis of genetic or metabolic causes is crucial for determining treatment options, accurate prognosis, and counselling for patients with CP. Mu et al. [5] identified three commonly used therapies for children with cerebral palsy: botulinum toxin, constraint-induced movement therapy (CIMT), and acupuncture. Botulinum toxin is frequently employed to relieve spasticity and dystonia, thereby increasing range of motion and functional abilities in children diagnosed with CP [7]. CIMT represents an evolving methodology and promotes greater utilization of affected upper limbs spontaneously while reducing the consequences of acquired disuse [8]. In some regions, e.g., in Asia, acupuncture is widely adopted, purportedly prompting stimulation within the cerebral cortex and peripheral nerves and consequently facilitating reductions in muscle tension [9].

In addition to the previously mentioned therapies, gait therapy has emerged as a crucial element for individuals with CP [10,11]. Advances in robotics and computer technology have stimulated collaboration among engineers, physicians, and physiotherapists to create equipment capable of facilitating therapeutic interventions under dynamic weight support conditions, thereby promoting an independent gait. Among these innovations, the Lokomat is an example of a robotic exoskeleton designed to support gait rehabilitation [12,13]. The scientific underpinning of robot-assisted gait training is rooted in spinal automatism (central pattern generators), central nervous system plasticity, and motor learning [14,15]. Numerous studies have demonstrated the effectiveness of robot-assisted locomotor therapy in improving gait among adult stroke survivors, those with brain or spinal injuries [16,17], and individuals with other neurological conditions, including Parkinson’s disease [18] and multiple sclerosis [19]. In addition, this therapy positively affected postural alignment, cardiovascular health, muscle metabolism, bowel motility, tissue health, and overall quality of life [16]. However, despite these benefits, Pawłowski et al. [13] highlighted the lack of specific guidelines for Lokomat training, noting the absence of consensus on optimal training parameters such as duration, intensity, and patient weight support levels. Meyer-Heim et al. [20] conducted an average of 15.1 ± 4.1 training sessions, during which patients walked an average distance of 842 ± 291 m per session, with each session lasting approximately 31.5 ± 7.1 min. Borggraefe et al. [21] implemented therapy sessions with an average duration of 34 min. The mean walking distance per session was 927 m. Over the course of the 3-week trial, the total walking distance reached 11.129 m. Moreover, the authors reported that body weight unloading initially started at 50% and was gradually reduced to nearly zero over the course of the therapy sessions, without causing significant knee buckling. Additionally, a 50% leading force was applied using the impedance control strategy. Despite these limitations, observational assessments and patient-reported outcomes provide valuable insights into the impact of Lokomat training on functional independence and quality of life for children with cerebral palsy. Some authors [20,21,22,23] have demonstrated the positive effects of Lokomat training on functional capacity levels in CP patients, although they have not explored trends in various parameters recorded by the Lokomat. This study aimed to investigate the influence of Lokomat training on gait parameters and discern trends in the parameters applied and assessed during training.

## 2. Materials and Methods

### 2.1. Participants’ Characteristics

This study was conducted in the Park Zdrowia Modern Rehabilitation Centre in Kampinos. The study included 26 ambulatory patients (13 male and 13 female individuals) with CP (Table 1).

The inclusion criteria were participant age from 3 to 24 years; diagnosis of cerebral palsy; ability to independently perform the Timed Up and Go test (TUG) and 10 m walk test (10mWT test), including the use of orthopaedic equipment; classification I–III according to the Gross Motor Function Classification System (GMFCS) [24]; understanding of instructions and verbal commands; physical disposition to begin a cycle of sessions on the Lokomat; ability to report any pain or discomfort that might occur during therapy; and no previous experience with the robot-assisted gait training.

Exclusion criteria encompassed conditions that might hinder locomotor training or present physical limitations upon using the robotic device. According to the Lokomat manufacturer’s manual (Hocoma AG, Volketswil, Switzerland), children should be excluded if they have severe lower-extremity muscle contractures (>20° knee extension deficit, >40° hip extension deficit), hip instability/subluxation >45%, received botulinum toxin A injections to lower limbs within the last three months, undergone neurosurgical or orthopedic interventions in the lower limbs within the nine months preceding therapy with Lokomat, an uncontrolled seizure disorder, vascular disorders of the lower limbs or have open skin lesions, or if they are unable to cooperate or be adequately positioned within the Lokomat [25].

The Gross Motor Function Classification System (GMFCS) was used to assess the motor skills of children with CP. The GMFCS consists of five levels, briefly described as follows: Level I—the patient moves without limitations; Level II—the patient moves with some restrictions on uneven surfaces, inclines, and in crowded areas; Level III—the patient walks indoors or outdoors on a level surface with the assistance of a mobility device or manually propels a wheelchair; Level IV—the patient may walk short distances with a walker or increasingly rely on wheeled mobility aids at home and in the community. Those classified at Level V experience significant limitations in antigravity movement, with all areas of motor function being restricted [24].

Consequently, the patients participating in the study were representatives of the following levels: Level I (4 people), Level II (12 people), and Level III (10 people). As per the GMFCS scale, these individuals could walk with or without manual support devices. While six subjects did not require orthopaedic assistance in their daily activities, the majority used assistive devices such as crutches (1 person), a tripod (3 persons), an anterior walker (7 participants), and a posterior walker (9 participants) (Table 1).

### 2.2. Ethical Approval

The study protocol was approved by the Institutional Review Board of Józef Piłsudski University of Physical Education in Warsaw, Poland (protocol code SKE01-15/2023 and date of approval 24 March 2023). This work was carried out in accordance with the Declaration of Helsinki. Informed consent was obtained from all subjects involved in the study aged over 16 and/or their parents (for patients under 18).

### 2.3. Devices, Procedures and Outcome Measures

#### 2.3.1. Lokomat Training Sessions

**Figure 1:**
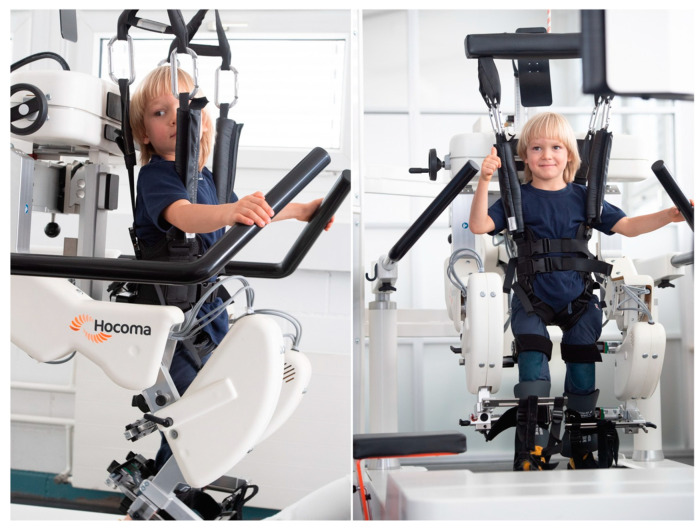
The Lokomat Pro 6 (Hocoma AG, Volketswil, Switzerland) (picture provided from the manufacturer’s resources).

The Lokomat Pro 6 (Hocoma AG, Volketswil, Switzerland) is a driven gait orthosis designed for intensive and repetitive gait therapy, suitable for children and adults (Figure 1). This device comprises an exoskeleton adaptable to individual patients, a dynamic body weight support system, and a treadmill [26]. During the 4-week standard rehabilitation program, the children participated in Lokomat training sessions (Table 1). The duration of Lokomat therapy was capped at 45 min, though the length of training varied based on the patient’s capabilities. Before the first training session of each cycle, the Lokomat, with its orthosis, was adjusted to fit the individual. This adjustment aimed to provide comfortable training conditions, following the manufacturer’s instructions (Hocoma AG, Volketswil, Switzerland). Workout parameters were tailored to each patient’s abilities and progress, ensuring the ability to maintain speed while providing tracking feedback on the screen. The supervising therapist encouraged active participation and proper positioning throughout the sessions.

The analysis included parameters of training sessions from Lokomat, all defined and stored in the system, enabling comparison over time within a patient. These parameters included (1) session time [min, s]—the duration of a single training session, with a default duration of 45 min. The duration could change depending on the patient’s disposition or fatigue; (2) walking distance [m]—the distance covered during a training session; (3) number of steps—the number of steps during a single training session; (4) body weight support (BWS) [%]—the amount of the offloading of the patient’s body weight; (5) guidance force (GF) [%], which determines the degree to which the patient’s movements are guided by the Lokomat orthoses during walking; and (6) mean walking speed [km/h], which determines the mean velocity of the treadmill, thus influencing the patient’s gait speed. This value is adjustable from 0.5 to 3.2 km/h [26,27].

#### 2.3.2. 10 m Walk Test (10mWT) and Timed Up and Go (TUG) Test

The measurements for the 10 m walk test (10mWT) and Timed Up and Go (TUG) test were taken on both the first and last day of the rehabilitation process, once each, in the following sequence: 10mWT, followed by TUG. Patients rested in a seated position for 3 min between tests. The time of the tests was recorded in seconds with precision to the hundredth of a second, using the Kalenji ON Rhythm 110 (Decathlon, Paris, France) digital stopwatch. Before the test, an experienced therapist provided instructions on conducting the test. Running during tests was not allowed.

The 10 m walk test (10mWT) is a clinical assessment used to measure the time it takes for an individual to walk a distance of 10 m in a straight line. The time was counted from the command “start” until the moment the ten-meter line was crossed.

**Figure 2:**
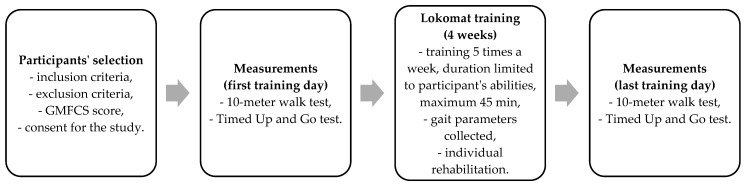
The study protocol.

The Timed Up and Go (TUG) test is a clinical assessment used to evaluate mobility and functional mobility in individuals. At the beginning of the test, the patient sat on a chair with feet on the floor. On the command “start”, the patient raised and covered a distance of 3 m in a straight line, turned and walked back to the chair, and sat down. Both tests are commonly used in rehabilitation to assess changes in walking ability over time or in response to interventions [28,29]. The full protocol is described in Figure 2.

### 2.4. Statistical Analysis

Statistical analysis was performed using Statistica v. 12 (TIBCO Software, Inc., Palo Alto, CA, USA). The threshold for statistical significance was set at *p* < 0.05. Wilcoxon’s test was used for the 10mWT and TUG results separately in the female and male groups, as well as in the whole group.

The minimum and maximal values for each parameter measured by Lokomat during therapy were exported for each group, i.e., females, males, and the entire cohort. Wilcoxon’s test was then applied to assess the statistical significance of these values. Additionally, the percentage increase or decrease resulting from Lokomat training in each parameter was calculated, thus elucidating its effect.

**Figure 3:**
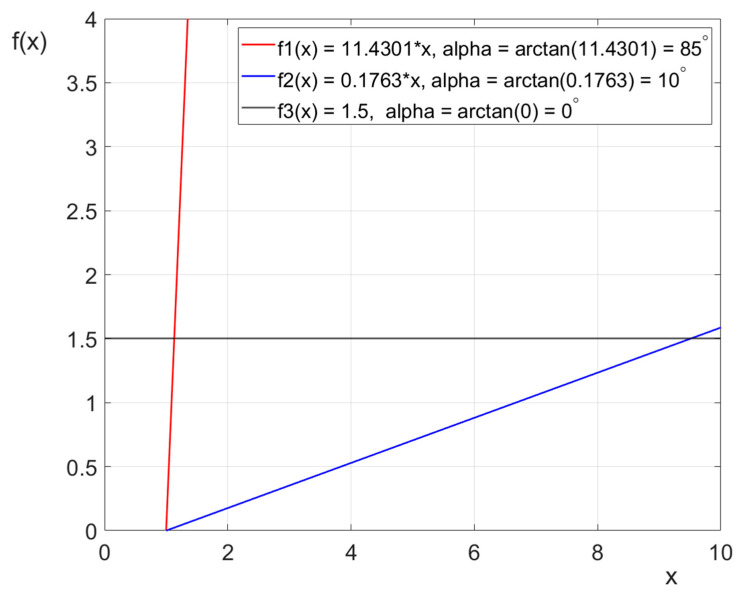
An example of three linear trend functions without free expression demonstrates how the slope of their graphs changes based on the value of the directional coefficient.

The last step involved the trend analysis. A time progress curve was plotted for each Lokomat parameter for each individual separately. Following this, a linear trend function of the form *f*(*x*) *= ax + b* was determined, where *a* represents the directional coefficient, indicating the slope of the line, and *b* represents the y-intercept [30]. The directional coefficient of the function equals the tangent of the angle (*alpha*) formed by the slope of the function’s graph with the abscissa (horizontal) axis. Thus, *a = tan* (*alpha*). Calculating the angle of alpha (*alpha* = *arctan* (*a*)) made it possible to show what the trend was, i.e., increasing (if the values of the angle were positive) or decreasing (if the values of the angle were negative), and how strong the trend was (the bigger the alpha, the faster the line goes up) (Figure 3).

## 3. Results

### 3.1. 10 m Walk Test (10mWT) and Timed Up and Go (TUG) Test

The median duration of the 10 m walk test significantly (*p* = 0.001) decreased in the whole group and in the male and female groups by 1.12 s (~4.99%), 2.81 s (~11.3%), and 0.76 s (~4%), respectively. The median duration of the TUG test decreased significantly in all the above-mentioned groups by 1.67 s (~4.01%), 0.06 s (~0.16%), and 3.61 s (~7.97%). The results of the 10mWT and TUG are presented in Table 2.

### 3.2. Persistent Trends in Lokomat Therapy

The analysis covered six Lokomat parameters, capturing their minimum and maximum values (Table 3). Across all subjects, a consistent pattern emerged. Typically, the minimum values were achieved during the initial training session, while the maximum values were consistently observed during the final or one of the penultimate meetings. This trend was the opposite for the body weight support and guidance force values.

Examining the difference between the median of the minimum and maximum values, it is evident that within the female group, the smallest increase occurred in session time, registering at 1.73%. The situation was similar in the male group (3.45%) and the entire cohort (2.48%). The highest increases, amounting to 61.86% and 41.94%, were observed in the male group and the entire cohort, respectively, for the number of steps taken. Notably, the median percentage of body weight support decreased by 42.91% in the male group, 39.24% in the female group, and 40.59% across the entire group.

The findings outlined in Table 4 support the preceding analysis (Table 3). Across the male group, the female group, and the entire cohort, the linear trend curves for distance and number of steps exhibited near perpendicular alignment with the horizontal axis. This alignment suggests significant improvement in these parameters, with values showing a consistent upward trend, as evidenced by the positivity and high magnitude of values in both the first and third quartile. In contrast, a consistent trend was noted for speed, with the trend line aligned parallel with the horizontal axis. The median slope angle of the trend line ranged from 1.6° in the female group to 1.92° in the male group. Decreasing trends were observed for body weight support and guidance force. The slope angles of the trend lines for body weight support averaged between −12.52° for females and −13.93° for males. Similarly, guidance force exhibited a monotonic decline, with median slope angles of −30.75° for males and −35.75° for females.

## 4. Discussion

This study aimed to examine the impact of Lokomat training on gait parameters and identify trends in the parameters used and evaluated during training. The study comprised 13 male and 13 female individuals, all exhibiting similar levels on the GMFCS scale. Within both groups, two individuals were classified at Level I, six at Level II, and five at Level III. Although the male group was older, taller, and heavier, and male individuals also used different number of walking aids compared to the girls (anterior walker (male individuals: 5, female individuals: 2), a tripod (male: 1, females: 2), crutches (male: 1, female: 0), a posterior walker (male individuals: 3, female individuals: 6), and no walking aid (male individuals: 3, female individuals: 3), there were no other significant differences between the groups in the study results. Therefore, the subsequent discussion will be concerned with the entire group’s results.

The results of the 10mWT and TUG tests revealed significant decreases following Lokomat therapy, with an approximately 4.99% decline in the 10mWT and a 4.01% decline in the TUG for the entire group. These findings are consistent with those reported in previous studies [13,20,21,31,32], although the decrease in values observed in the cited studies was notably higher. Likewise, the values for both the pre- and post-therapy tests were considerably lower in the cited papers than in the present study, suggesting that the subjects in those studies exhibited faster performance.

According to van Dellen and Labruyère [11], Lokomat therapy seems to be equally as effective as standard therapy approaches such as over-ground walking or manual treadmill therapy. One reason for this might be that the most commonly adjusted parameters—including the regulation of gait speed, the amount of unloading (referred to as body weight support), and a scaling factor for the force field that maintains the legs on the desired spatiotemporal trajectory (referred to as guidance force)—are not optimally used [33]. Talaty and Esquenazi [34] similarly underscore the importance of in-hospital rehabilitation for patients following an acute stroke. They highlight that both Lokomat and conventional gait training (CGT) significantly enhance functional outcomes as evidenced by improvements in measures such as the Functional Independence Measure and the 10 m walk test. The authors showed that the CGT group benefited from a greater number of therapy sessions than the Lokomat group. This suggests that while both interventions are effective, Lokomat may provide a more efficient approach to gait retraining by delivering a higher therapeutic “dose” despite requiring fewer sessions.

In the present study, the consistent trend was for speed, with the trend line aligned almost parallel with the horizontal axis. The slope angle of the trend line was low, measuring 1.79° across the whole group. However, the median speed during Lokomat training increased by only 25%. Koenig et al. [35] demonstrated that increasing gait speed raises the heart rate, thus intensifying therapy. Moreover, van Kammen et al. [36] showed that increasing gait speed in patients with cerebral palsy enhances muscle activation. Nevertheless, some evidence supports reducing walking speed to activate supra spinal centres [37,38]. Van Dellen and Labruyère [11] conducted a comprehensive review revealing diverse strategies for body weight support. Most studies maintained weight support below 50% [39,40,41], while others consistently kept it at or above this threshold [42,43,44]. In the present investigation, body weight support initially had median value 64.11%, decreasing to 45.6% by therapy’s end. Notably, existing evidence underscores that reducing body weight support can heighten metabolic costs [45] and elevate heart rates [35], attributed to increased muscle activation [36,43,44]. The setting of guidance force represents the final parameter adjusted during training sessions. While some therapists advocate for minimizing robot–patient interactions, favouring a low guidance force [42], others advocate resistance training with the Lokomat [31,46]. In the present study, the guidance force had a consistent decline, with median slope angles of −33.43°. This downward trend resulted in a notable 23.89% difference between initial and final values. Studies by van Kammen et al. [36,47], demonstrated that reducing guidance force can heighten muscle activation. Similarly, Krishnan et al. [42] showed that during trajectory tracking tasks in the Lokomat, employing a low guidance force can enhance muscle activation and diminish tracking errors.

The last that should be discussed is the number of steps and distance covered during the Lokomat training. In the present study, the linear trend curves for distance and number of steps displayed nearly perpendicular alignment to the horizontal axis, measuring at 87.78° and 88.74°, respectively. This alignment strongly indicates significant enhancements in these metrics, with values consistently trending upward. This notion is supported by a substantial 39.38% increase in distance covered and a 41.94% increase in the number of steps observed after the rehabilitation treatment. These findings not only highlight the effectiveness of Lokomat training in promoting increased mobility but also emphasize the potential of this therapy to facilitate meaningful improvements in gait parameters for children with cerebral palsy.

This study has some limitations that should be mentioned. Firstly, the study did not include a control group, which would have allowed for a more robust comparison between Lokomat therapy and standard rehabilitation approaches. Without a control group, it is challenging to attribute the observed improvements solely to Lokomat therapy, as other factors, such as natural progression or concurrent therapies, could also have contributed to the outcomes. It must be pointed out that it was not possible to blind the study due to the nature of Lokomat training. The second limitation of this study is the relatively small sample size, which may have limited the generalizability of the findings. This study included 26 children affected by cerebral palsy with different ages and GMFCS levels, which, while sufficient for an exploratory investigation, may not fully capture the diversity of responses to Lokomat therapy across different demographic and clinical profiles. Furthermore, the study duration was limited to a 4-week rehabilitation program, which may not capture the full extent of long-term effects or potential plateauing of benefits over time. Future studies with larger sample sizes, randomized controlled designs, and longer follow-up periods would provide more definitive insights into the efficacy and optimal parameters of Lokomat therapy for children with cerebral palsy.

## 5. Conclusions

This study identified a consistent trend of increasing gait speed during therapy, albeit at a modest rate. While the literature suggests that higher speeds may intensify therapy, contradictory evidence suggests benefits in reducing speed to activate certain neural centres. Body weight support exhibited a decreasing trend throughout therapy, potentially influencing metabolic costs and muscle activation. Similarly, guidance force decreased steadily during sessions, with evidence suggesting its impact on muscle activation and tracking accuracy. Furthermore, significant enhancements were noted in the distance covered and number of steps during Lokomat training, indicating positive progress in locomotor capabilities. Overall, this study contributes insights into the efficacy of Lokomat therapy and underscores the importance of optimizing adjustable parameters to maximize therapeutic benefits. Further research is warranted to explore individualized approaches and long-term outcomes in diverse patient populations.

## Figures and Tables

**Table 1:** Summary of patients’ anthropometric data: mean ± standard deviation, extreme values, and median with quartile distribution.

Group	Age [Years]	Body Height [cm]	Body Weight [kg]	BMI [kg/m^2^]	Number of Training Days on Lokomat	GMFCS ^1^ Scale/Number of Persons	Walking Aids/Number of Persons
Female individuals (*n* = 13)	9 ± 3.72 (4–17) 8 (7; 12)	125.92 ± 15.77 (106–158) 115 (110; 124)	28.62 ± 7.39 (19–40) 20 (18; 25)	17.96 ± 2.90 (16–23.6) 16.9 (16.10; 19.10)	15.77 ± 5.95 (10–26) 22 (11; 30)	Level I: 2 Level II: 6 Level III: 5	Anterior walker: 2 Tripod: 2 Crutches: 0 Posterior walker: 6 No: 3
Male individuals (*n* = 13)	12.08 ± 6.59 (5–23) 10 (7; 17)	145.15 ± 21.64 (114–175) 130 (120; 167)	41 ± 13 (25–65) 30 (21; 36)	19.02 ± 1.59 (16.7–21.7) 19.2 (17.80; 20)	21.46 ± 10.49 (10–42) 12 (11; 20)	Level I: 2 Level II: 6 Level III: 5	Anterior walker: 5 Tripod: 1 Crutches: 1 Posterior walker: 3 No: 3
Total (*n* = 26)	10.5 ± 5.47 (4–23) 8 (7; 14)	135.2 ± 20.98 (106–175) 120 (113; 145)	34.8 ± 12.13 (19–65) 23 (18; 35)	18.5 ± 2.37 (16–23.6) 18.05 (16.9; 20)	18.62 ± 8.84 (10–42) 15.50 (11; 24)	Level I: 4 Level II: 12 Level III: 10	Anterior walker: 7 Tripod: 3 Crutches: 1 Posterior walker: 9 No: 6

^1^ GMFCS—Gross Motor Function Classification System.

**Table 2:** Summary of TUG and 10mWT test results (median with quartile distribution) before and after therapy with the robot-assisted Lokomat orthosis in the whole group, the male group, and the female group, separately.

Parameter	Baseline	Post-Intervention	*p*-Value/ Decrease Percentage
The whole group (*n* = 26)			
TUG [s]	41.65 (21.56; 50.93)	39.98 (21.52; 48.87)	*p* = 0.001/4.01%
10mWT [s]	22.43 (15.87; 35.03)	21.31 (15.59; 31.18)	*p* = 0.001/4.99%
Male individuals (*n* = 13)			
TUG [s]	38.31 (21.56; 52.65)	38.25 (21.52; 51.16)	*p* = 0.001/0.16%
10mWT [s]	24.87 (17.06; 42.69)	22.06 (16.86; 41.86)	*p* = 0.001/11.3%
Female individuals (*n* = 13)			
TUG [s]	45.32 (33.08; 47.55)	41.71 (32; 45.73)	*p* = 0.001/7.97%
10mWT [s]	19 (15.62; 24.12)	18.24 (15.10; 22.69)	*p* = 0.001/4%

**Table 3:** Median with quartile distribution of minimum and maximum parameter values exported from Lokomat sessions during therapy with Lokomat robot-assisted orthosis, analyzed separately for the whole group, the male group, and the female group.

Parameter	Male Individuals (*n* = 13)		*p*-Value/Increase Percentage	Female Individuals (*n* = 13)		*p*-Value/Increase Percentage	The Whole Group (*n* = 26)		*p*-Value/Increase Percentage
	Min	Max		Min	Max		Min	Max	
Distance [m]	850.72 (661.57; 978.23)	1135.16 (1121.52; 12.05)	*p* = 0.001 30.78%	886.42 (766.31; 947.85)	1159.27 (1058.03; 1211.76)	*p* = 0.001 33.44%	830.55 (661.57; 978.23)	1157.59 (1065.15; 1205.32)	*p* = 0.001 39.38%
Distance [steps]	1636 (1418; 2142)	2648 (2132; 2770)	*p* = 0.001 61.86%	1758 (1440; 1946)	2314 (2278; 2762)	*p* = 0.001 31.63%	1705 (1418; 2038)	2420 (2176; 2762)	*p* = 0.001 41.94%
Session time [min]	44.02 (35.88; 45)	45.54 (45.18; 45.61)	*p* = 0.001 3.45%	44.52 (34.66; 44.98)	45.29 (45.19; 45.56)	*p* = 0.001 1.73%	44.27 (34.66; 45)	45.37 (45.18; 45.61)	*p* = 0.001 2.48%
Mean BWS [%]	44.86 (43.64; 48.79)	64.11 (58.97; 72.78)	*p* = 0.001 42.91%	46.92 (43.05; 52)	65.33 (55.38; 79.1)	*p* = 0.001 39.24%	45.6 (43.05; 51.72)	64.11 (57.76; 77)	*p* = 0.001 40.59%
Mean GF [%]	80.86 (78.89; 88.98)	100 (100; 100)	*p* = 0.001 23.67%	78.79 (73.76; 87.47)	100 (100; 100)	*p* = 0.002 26.92%	80.72 (73.76; 88.98)	100 (100; 100)	*p* = 0.001 23.89%

BWS—body weight support, GF—guidance force.

**Table 4:** Median with quartile distribution of the slope angle of the linear trend curve for parameters exported from Lokomat sessions during therapy with the Lokomat robot-assisted orthosis, analyzed separately for the whole group as well as male and female individuals.

Parameter	Median (First Quartile, Third Quartile) [deg]		
	Male Individuals (*n* = 13)	Female Individuals (*n* = 13)	The Whole Group (*n* = 26)
Distance [m]	87.68 (87.02; 88.86)	87.89 (87.33; 88.65)	87.78 (87.07; 88.68)
Distance [steps]	88.73 (87.25; 89.33)	88.74 (88.42; 89.39)	88.74 (87.25; 89.37)
Session time [min]	2.15 (0.61; 34.67)	0.32 (−0.29; 43.5)	1.88 (0; 36.7)
Mean speed [km/h]	1.92 (1.25; 3.05)	1.62 (0.80; 2.32)	1.79 (0.80; 2.59)
Mean BWS [%]	−13.93 (−29.21; −6.33)	−12.52 (−39.76; 16.69)	−13.52 (−36.46; 6.11)
Mean GF [%]	−30.75 (−52.54; 5.89)	−35.75 (−53.67; −11.14)	−33.43 (−53.33; −0.86)

BWS—body weight support, GF—guidance force.

## Data Availability

The measurement data used to support the findings of this study are available from the corresponding author upon request.
